# Crown-Ether-Modified SBA-15 for the Adsorption of Cr(VI) and Zn(II) from Water

**DOI:** 10.3390/ma14175060

**Published:** 2021-09-03

**Authors:** Jong-Man Yoo, Sung Soo Park, Yong-Zhu Yan, Chang-Sik Ha

**Affiliations:** Department of Polymer Science and Engineering, School of Chemical Engineering, Pusan National University, Busan 46241, Korea; dbaks124@gmail.com (J.-M.Y.); nanopss@pusan.ac.kr (S.S.P.); yzyancam@163.com (Y.-Z.Y.)

**Keywords:** mesoporous silica, crown ether, Cr(VI), Zn(II), selective adsorption

## Abstract

Recently, the release of some metal ions to the environment has been observed to cause serious damages to human health and the environment. Herein, a chromium(VI)- and zinc(II)-selective adsorbent (CB18crown6/SBA-15) was successfully fabricated through the covalent attachment of 4′-carboxybenzo-18-crown-6 (CB18crown6) as a ligand on mesoporous silica support (SBA-15). The CB18crown6/SBA-15 adsorbent was characterized by Fourier-transform infrared (FTIR) spectrometry, X-ray diffraction (XRD), N_2_ adsorption–desorption, thermogravimetric analysis (TGA), scanning electron microscopy (SEM), and transmission electron microscopy (TEM). To evaluate its ability to selectively capture Cr(VI) and Zn(II), adsorption experiments were conducted. The influences of pH, initial concentration of metal ions, and coexisting metal ions on the adsorption process were examined. The CB18crown6/SBA-15 selectively adsorbed Cr(VI) at pH 2 and Zn(II) at pH 5, respectively, from the mixed aqueous solutions of chromium, zinc, lithium, cadmium, cobalt, strontium, and cesium ions. The data for the adsorption of Cr(VI) onto the CB18crown6/SBA-15 were well explained by the Langmuir adsorption isotherm. In addition, the recycling and reuse of CB18crown6/SBA-15 was successfully achieved, and 71 and 76% reuse efficiency of Cr(VI) and Zn(II), respectively, was obtained after five cycles. This study suggests that the use of the CB18crown6/SBA-15 can be a feasible approach for the selective remediation of Cr(VI) and Zn(II) contamination.

## 1. Introduction

When industrial water containing metal ions and heavy metal ions is released into nature, the contaminated water causes damages to various lifeforms, and eventually affects humans [1,2]. For instance, zinc can cause abdominal pain, vomiting, and headache, and is considered to be hazardous to human health and the environment [3], while the presence of hexavalent chromium (Cr(VI)) in water may cause ailments such as skin allergy, bronchial and liver damage, and kidney failure due to its frequent occurrence and toxicity to living organisms [3]. Thus, removal of metal ions from contaminated water is essential. Over the decades, several methods have been devoted to removing metal ions from aqueous solutions via various techniques, such as adsorption, ion exchange, sedimentation, and membrane processes [4,5,6,7]. Amongst them, adsorption is a convenient technique to remove metal ions from aqueous solutions, with the advantages of low cost, high efficiency, easy operation, and low secondary pollution impact [8].

Particularly, SBA-15—one of the most popular mesoporous silicas—has a large specific surface area (~1000 m^2^g^−1^), high porosity, and high hydrothermal and mechanical stability [9,10,11,12]. Thus, SBA-15 is an attractive material in effectively anchoring the metal ions due to the structural advantages [13,14]. However, SBA-15 does not provide fast adsorption and selectivity to metal ions, due to the lack of corresponding active sites [15,16]. Thus, to endow the SBA-15-based adsorbents with selective removal capability of metal ions, the surface modification must be considered. Fortunately, its surface is abundant with silanol groups that can be used as reaction sites to functionalize it.

Most crown ethers, having polarizing holes and outer hydrocarbons, are suitable for the adsorption of metal ions [17,18]. The oxygen ring present in the crown ether with electron-rich ring structures could selectively and effectively form stable complexes with metal ions, with compatible dimensions and strong affinity [17,18,19,20]. Moreover, the adsorption of metal ions can be easily controlled through the adjustment of pH. Luo et al. [20] and Mohamed et al. [21] reported the adsorption of metal ions (Sr^2+^, Cs^+^, Li^+^) using crown ether molecules or polybenzoxazine functionalized with crown ether moieties as adsorbents. In the case of using crown ether moieties, it is difficult to separate the adsorbent from the solution and the organic polymers as a support due to their chemically unstable nature. Meanwhile, Duman et al. [17] reported the adsorption of metal ions (Cr^3+^, Co^2+^, Ni^2+^) using activated carbon with crown ether molecules introduced by electrostatic interaction as an absorbent. Hong et al. [19] reported the adsorption of Ag^+^ using mesoporous silica with crown ether moieties introduced by chemical reaction. These works mainly studied the adsorption behavior using a single solution of metal ions.

In particular, highly selective adsorption of Cr(VI) and Zn(II) in aqueous solutions with various metal ions using an adsorbent chemically modified with crown ether moieties on mesoporous silica with high surface area and uniform pore size have not yet been reported, to the best of our knowledge. Easy separation the adsorbent from the solution is very important, as is highly selective adsorption of metal ions. Meanwhile, if improved for the low-cost synthesis of the adsorbent material, the commercial application value will be much higher.

In addition, most crown ethers do not contain functional groups, so it is difficult to graft them to other materials by covalent bonding [18,20,21,22]. For instance, Awual et al. [18] reported that the fabrication of a mesoporous adsorbent by direct physical immobilization of dibenzo-30-crown-10-ether (DB30C10) to a mesoporous silica substrate. However, crown ethers mixed with inorganic mesoporous silica without covalent bonding, but simply via physical blending, are likely to cause the elution of effective crown ether during the adsorption process. Thus, the choice of a crown ether is quite important for effectively introducing it to SBA-15. 4′-Carboxybenzo-18-crown-6 containing a carboxyl group was selected in this work, since a dehydration condensation reaction occurs with the amino group to form an amide bond under the action of the activator. The SBA-15 was modified with (3-aminopropyl) triethoxysilane (APTES) to make the surface of SBA-15 contain an amino group. Thanks to the choice of modifying agent, the amines present in the modified SBA-15 (SBA-15-NH_2_) can combine well with the carboxyl group. For more effective bonding of the carboxyl group with the amino group, 4′-carboxybenzo-18-crown-6 was added with N-hydroxysuccinimide (NHS) and 1-(3-dimethylaminopropyl)-3-ethylcarbodiimide hydrochloride (EDC) to enable the carboxyl group to change into the initiating mode and then react effectively with the amino group on the SBA-15-NH_2_.

In this work, we employed SBA-15 as a supporting material, for which carboxy-containing 4′-carboxybenzo-18-crown-6-ether (CB18crown6) was grafted onto the surface of the SBA-15-NH_2_ (amino-functionalized SBA-15) through a facile and robust covalent grafting approach. This led to CB18crown6/SBA-15 with surface-accessible crown ether. The structural characteristics of the CB18crown6/SBA-15 adsorbent before and after modification, as well as its adsorption behavior, were investigated. Batch adsorption experiments were conducted to study the influences of pH, initial metal ion concentrations, and coexisting metal ions on the adsorption process. The selectivity of the CB18crown6/SBA-15 adsorbent towards Cr(VI) and Zn(II) ions was evaluated in the presence of coexisting metal ions of chromium, zinc, lithium, cadmium, cobalt, strontium, and cesium ions.

## 2. Materials and Methods

### 2.1. Materials

Poly(ethylene oxide)-block-poly(propylene oxide)-block-poly(ethylene oxide) (PEO_20_PPO_70_PEO_20_: P123, Molecular weight (MW) = 5800), tetraethyl orthosilicate (TEOS, 98%), 3-aminopropyltriethoxysilane (APTES, ≥98%), N-hydroxysuccinimide (NHS, 98%), tetrahydrofuran (THF, 99.9%), toluene, cadmium nitrate tetrahydrate (CdN_2_O_8_·4H_2_O, ≥98%), cobalt(II) nitrate hexahydrate (Co(NO_3_)_2_·6H_2_O, ≥98%), cesium nitrate (CsNO_3_, 99%), zinc nitrate hexahydrate (Zn(NO_3_)_2_·6H_2_O, ≥99%), strontium nitrate (Sr(NO_3_)_2_, 99%), and lithium nitrate (LiNO_3_, 99%) were purchased from Sigma-Aldrich (St. Louis, MO, USA). 4′-Carboxybenzo-18-crown-6-ether (Crown, >97%) and 1-(3-dimethylaminopropyl)-3-ethylcarbodiimide hydrochloride (EDC, 98%) were purchased from Tokyo Chemical Industry (Tokyo, Japan). Hydrochloric acid and sodium hydroxide were purchased from DaeJung Chemicals & Metals Co., Ltd. (Siheung, Korea). Anhydrous ethanol (99%) was purchased from Junsei Chemical (Tokyo, Japan). Potassium dichromate (K_2_Cr_2_O_7_, ≥99%) was purchased from Osaka Chemical (Osaka, Japan). All reagents were used without further purification [23].

### 2.2. Synthesis of Santa Barbara Amorphous-15 (SBA-15)

SBA-15 was prepared by a sol–gel reaction as described in our previous work, with optimized material ratios, as follows [23]: SBA-15 was synthesized using poly (ethylene glycol)-block-poly (propylene glycol)-block-poly (ethylene glycol) (P123) as a template and tetraethyl orthosilicate (TEOS) as a silica source. First, P123 (16.0214 g) was added to 500 mL of distilled water and stirred at 35 °C for 30 min. Then, hydrochloric acid (80 mL) was poured into the P123 aqueous solution, followed by dropwise addition of TEOS (36.87 mL). After stirring for an additional 1 h, the mixture was kept at 35 °C for 24 h. After raising the temperature to 100 °C, it was again kept for 24 h, then washed with distilled water 2–3 times, followed by washing with ethanol once. When the washing was complete, it was dried in an oven (JEIO TECH, Kimpo, Korea) at 80 °C for 24 h. This sample was named SBA-15-T.

Two methods were used to remove the template in order to compare the effectiveness of further modification, as well as the physicochemical properties of the SBA-15, since the surface functionalization with an amine group and further modification with crown ether are key steps to prepare the required adsorbents for this work. For calcination, 10 g of the SBA-15-T was calcined in an oven at 540 °C for 9 h [24]. For extraction, 1 g of the SBA-15-T was added to the mixture of hydrochloric acid (36.5%, 3 mL) and ethanol (150 mL), followed by stirring for 12 h at 60 °C [25]. The calcined sample was named SBA-15-Cal, while the extraction sample was named SBA-15-Ex.

### 2.3. Surface Functionalization of SBA-15

**Scheme 1:**
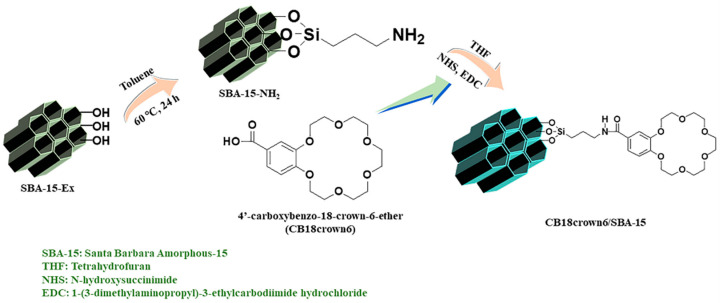
Schematic illustration of the preparation of CB18crown6/SBA-15.

The APTES-functionalized SBA-15 was prepared using the following method: 0.9 mL of APTES was added to 0.3 g of SBA-15-Ex in 50 mL of toluene, followed by stirring at 60 °C for 24 h. The sample was washed several times with toluene and then dried in a vacuum oven (JEIO TECH, Kimpo, Korea) at 60 °C for 24 h; this sample was named SBA-15-NH_2_ (Scheme 1). For comparison, SBA-15-Cal-NH_2_ was synthesized using a similar procedure.

### 2.4. Modification of SBA-15-NH_2_ with Crown Ether

To convert the carboxyl group in 4′-carboxybenzo-18-crown-6-ether into the initiation mode, the crown ether (0.01 g) was stirred and dispersed in THF (50 mL), and then EDC (0.5 mmol, 0.0958 g) and NHS (0.5 mmol, 0.0575 g) were slowly added and stirred for 1 h. SBA-15-NH_2_ (0.1 g) was suspended in a beaker containing THF (50 mL), and then it was added to the crown ether mixture and stirred for 24 h at room temperature. The final adsorbent was collected by centrifugation, and then washed with THF, and vacuum-dried at 60 °C for 24 h. This sample was denoted as CB18crown6/SBA-15 (Scheme 1). For comparison, CB18crown6/SBA-15-Cal was synthesized using a similar procedure.

### 2.5. Batch Adsorption Experiments

The adsorption of metal ions onto CB18crown6/SBA-15 was examined through batch adsorption experiments. In general, potentially toxic metal ions coexist in industrial wastewater, limiting its reusability for practical applications. Therefore, it is important to develop an efficient material featuring selective adsorption capacities for specific metal ions in multicomponent systems. Coexistent ions in multicomponent systems might begin to compete for the adsorption sites of CB18crown6/SBA-15. Therefore, to investigate the practical applicability of CB18crown6/SBA-15, we need to understand the role of each metal ion and its adsorption behavior. For this purpose, the seven metal ions designated in this study were chromium (Cr), cadmium (Cd), zinc (Zn), lithium (Li), cobalt (Co), strontium (Sr), and cesium (Cs). Competitive metal ion adsorption was performed in the pH range 2–6 in a batch experiment with a mixture of all seven metal ions at concentrations of 0.5 mmol/L (25 °C). In this study, the adsorption selectivity studies of Cr(VI) were mainly performed in highly acidic media, though such conditions are unusual for water treatment, based on two major factors: (1) the experimental results indicated that CB18crown6/SBA-15 exhibits effectively selective adsorption of Cr(VI) at pH 2, as in previous reports, which will be discussed later in more detail; (2) when the pH exceeded 7–8, metal ions were precipitated, so we did not measure the adsorption experiments with pH > 7. This may be because for Zn(II), Cd(II), and Co(II), the addition of NaOH to increase the pH may result in the generation of white precipitates of metal hydroxides or salts. Metal ion adsorption experiments were conducted by adding 0.01 g of CB18crown6/SBA-15 to 10 mL of the prepared metal ions in aqueous solution (Milli Q, Merck, Kenilworth, NJ, USA) with shaking for 24 h. After each adsorption experiment, the metal-ion-adsorbed CB18crown6/SBA-15 was separated by centrifugation from the solution. The amount of metal adsorbed by the adsorbent was measured by inductively coupled plasma optical emission spectrometry (ICP-OES). To investigate the improvement of the adsorption capacity of SBA-15 by introducing CB18crown6, isotherm adsorption experiments were carried out on SBA-15-NH_2_ and CB18crown6/SBA-15 by changing the initial Cr(VI) concentration from 1 to 50 mg/L at room temperature.

The adsorbed amount, q_e_ (mg/g), was estimated using the following equation:

(1) $$q_{e}=\frac{\left(C_{0}-C_{e}\right)\times V}{m}$$

where C_0_ (mg/L) is the initial concentration of a metal ion adsorbate, C_e_ (mg/L) is the equilibrium concentration of the metal ion adsorbate, V (mL) is the volume of the solution, and m (mg) is the mass of the adsorbent [26].

### 2.6. Characterization

Samples were characterized by Fourier-transform infrared (FTIR) spectrometry (FTIR-4100, JASCO, Tokyo, Japan) in the scanning range 400–4000 cm^−1^. Brunauer–Emmett–Teller (BET) specific surface area and Barrett–Joyner–Halenda (BJH) pore size distributions were determined using N_2_ adsorption–desorption isotherms (Micromeritics ASAP 2020 V3.04G, Micromeritics, Norcross, GA, USA). Thermogravimetric analysis was carried out on a PerkinElmer Pyris Diamond TG (PerkinElmer, Waltham, MA, USA) in a N_2_ atmosphere at a heating rate of 5 °C/min. The concentrations of metal ions were measured using an inductively coupled plasma optical emission spectrometer (ICP-OES-720, Agilent Technologies, Santa Clara, CA, USA). The X-ray diffraction (XRD, Bruker AXS, Billerica, MA, USA) was conducted using Cu Kα irradiation at 40 kV and 40 mA in the 2θ ranging from 1.2 to 10°. The morphologies of adsorbents were analyzed by field emission scanning electron microscopy (FESEM, ZEISS SUPRA 25 VP, Carl Zeiss AG, Jena, Germany) and transmission electron microscopy (TEM, JEOL 2011, Tokyo, Japan) at an acceleration voltage of 200 kV.

## 3. Results and Discussion

### 3.1. Characterization of Adsorbents

#### 3.1.1. FTIR Spectroscopic Analysis

**Figure 1:**
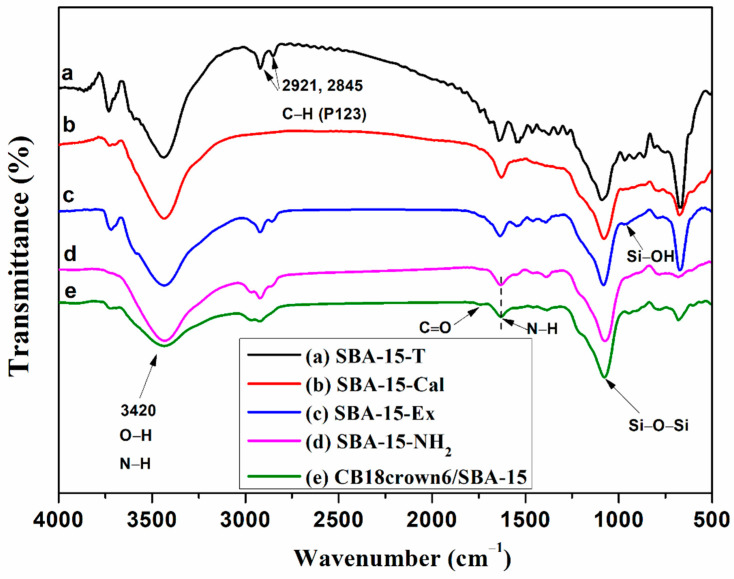
FTIR spectra of (**a**) SBA-15-T, (**b**) SBA-15-Cal, (**c**) SBA-15-Ex, (**d**) SBA-15-NH_2_, and (**e**) CB18crown6/SBA-15.

FTIR spectra were used to explore the changes in the functional group of SBA-15 after modifying. Figure 1a shows the FTIR spectrum of SBA-15 with a template, while Figure 1b,c show the FTIR spectra of the SBA-15-Cal and the SBA-15-Ex, respectively. For the SBA-15-T, the peaks at 2921 and 2845 cm^−1^ were due to the C–H stretching vibration of P123. As shown in Figure 1b,c, the peak due to P123 was totally absent after calcination (SBA-15-Cal), whereas it still remained after extraction (SBA-15-Ex), indicating the residuals of the silanol group in the SBA-15-Ex. It can also be seen that the peak at 962 cm^−1^ is significantly reduced after calcination but less reduced after extraction, which is associated with the silanol group (Si–OH) [27]. In this work, although the template P123 in SBA-15-Ex may not be completely removed, the remaining silanol groups on the surface of SBA-15-Ex may contribute to further functionalization or modification; thus, we chose SBA-15-Ex for the next step to synthesize the adsorbent. Figure 1d shows the FTIR spectrum of the SBA-15-NH_2_. The peak at 1625 cm^−1^ is due to the stretching vibrations of the N–H group [16]. In addition, the peaks identified in the SBA-15-Ex at 962 cm^−1^ due to the Si–OH groups were significantly decreased, indicating the successful modification of the SBA-15 by APTES. Figure 1e shows the FTIR spectrum of the SBA-15-NH_2_ modified with CB18crown6. The appearance of the peaks at 1738 and 1631 cm^−1^ suggests the presence of the C=O and N–H groups as CB18crown6 is attached to the SBA-15-NH_2_ surface. Compared with the intensity of the SBA-15-NH_2_, the intensity of the O–H and N–H vibration at 3420 cm^−1^ in CB18crown6/SBA-15 decreased. The FTIR results confirmed that CB18crown6 had been grafted onto the SBA-15-NH_2_. In addition, another peak for the Si–O–Si vibrational bond at 1081 cm^−1^ indicated that the structure of the mesoporous material remains intact during the modification process.

#### 3.1.2. XRD Analysis

**Figure 2:**
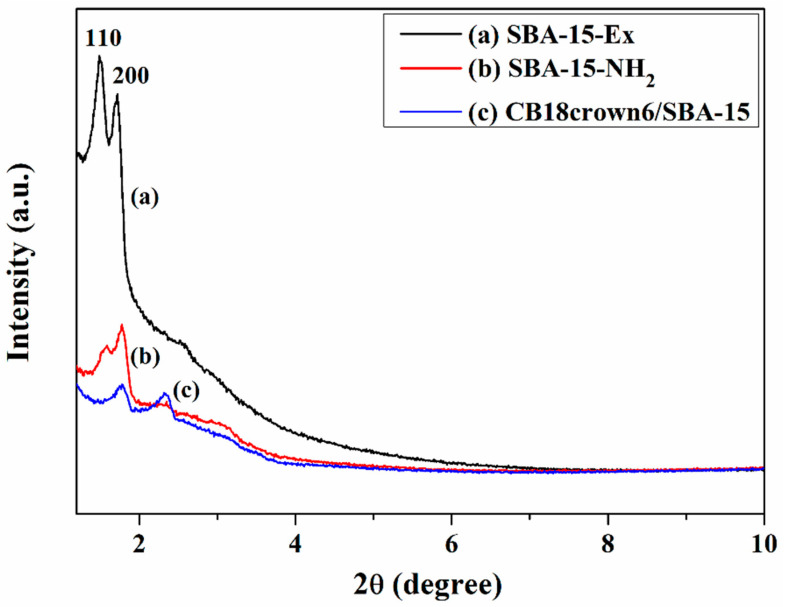
X-ray diffraction (XRD) patterns of (**a**) SBA-15-Ex, (**b**) SBA-15-NH_2_, and (**c**) CB18crown6/SBA-15.

The XRD patterns for SBA-15-Ex, SBA-15-NH_2_, and CB18crown6/SBA-15 are displayed in Figure 2. All of these samples exhibit two characteristic diffraction peaks in the range of 1.2°–2° that can be indexed to (110) and (200) diffraction associated with two-dimensional hexagonal symmetry (P6mm) [28,29]. The results indicate that after modification of SBA-15 with CB18crown6, CB18crown6/SBA-15 still shows ordered mesopores with uniform pore size. The intensities of these characteristic diffraction peaks decreased after grafting of APTES with respect to the SBA-15-Ex, and decreased further after the covalent attachment of CB18crown6. In addition, the position of these characteristic diffraction peaks shifted to the right after grafting of APTES, and shifted further after the covalent attachment of CB18crown6. The shift to the right indicates a decrease in the pore sizes. The decrease in intensity as well as the peak shifts indicate that the crown ether has been successfully introduced into SBA-15.

#### 3.1.3. Thermogravimetric Analysis

**Figure 3:**
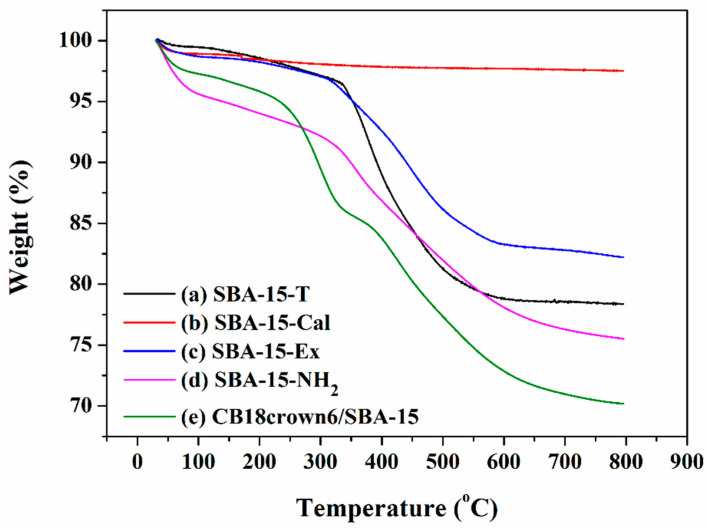
Thermogravimetric analysis curves of (**a**) SBA-15-T, (**b**) SBA-15-Cal, (**c**) SBA-15-Ex, (**d**) SBA-15-NH_2_, and (**e**) CB18crown6/SBA-15.

Figure 3 shows the TGA curves of SBA-15-T, SBA-15-Cal, SBA-15-Ex, SBA-15-NH_2_, and CB18crown6/SBA-15. Comparing Figure 3a–c, the SBA-15-T shows the greatest weight loss due to the degradation of the template P123 around 300 °C. The SBA-15-Cal in Figure 3b, where P123 was removed at high temperature by calcination, showed the highest thermal stability and no noticeable loss in weight. The weight loss up to around 100 °C resulted from the evaporation of the physisorbed water on the surface of the mesoporous materials. The SBA-15-Ex in Figure 3c displayed reduced weight loss compared to that of the SBA-15-T and greater weight loss than that of the SBA-15-Cal, due to the degradation of the remaining P123 and the silanol groups on the surface. Meanwhile, after grafting APTES onto the SBA-15-Ex, the weight loss of the SBA-15-NH_2_ increased to 25% due to the degradation of the organic moieties in SBA-15-NH_2_, owing to the presence of APTES. For CB18crown6/SBA-15, the weight loss further increased to 30% due to the decomposition of CB18crown6. It is notable that the residue contents of SBA-15-Ex, SBA-15-NH_2_, and CB18crown6/SBA-15 were 82.3, 75, and 70%, respectively, indicating that the content of APTES and CB18crown6 grafted to the surface of SBA-15-Ex was approximately 7.3 and 5%, respectively. These phenomena indicate the successful grafting of the CB18crown6 ligand onto the SBA-15.

#### 3.1.4. FESEM and TEM Analyses

**Figure 4:**
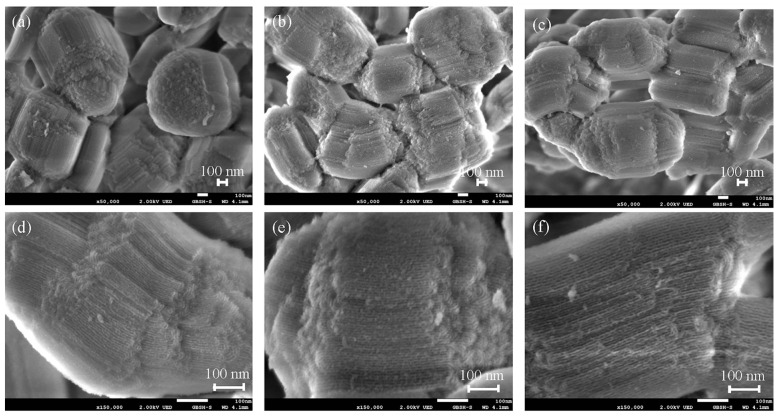
FESEM images of (**a**,**d**) SBA-15-Ex, (**b**,**e**) SBA-15-NH_2_, and (**c**,**f**) CB18crown6/SBA-15. (**a**–**c**): Low magnification; (**d**–**f**): High magnification.

**Figure 5:**
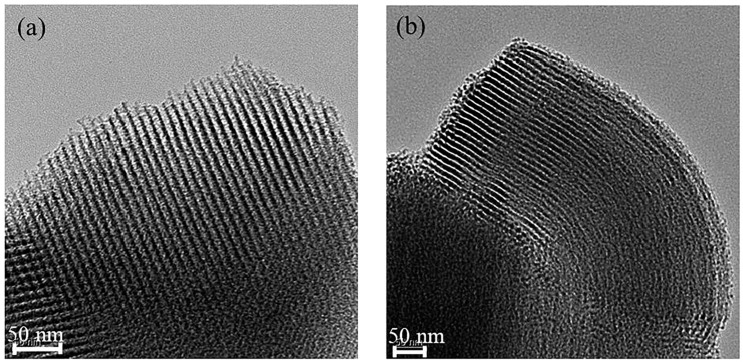
TEM images of (**a**) SBA-15-Ex and (**b**) CB18crown6/SBA-15.

The morphology and microstructure of the SBA-15-Ex, SBA-15-NH_2_, and CB18crown6/SBA-15 can be observed via FESEM (Figure 4). The synthesized SBA-15-Ex exhibited uniform, hexagonal, rod-like particles and uniform pore channels (Figure 4a,d). After modification, the morphologies of SBA-15-NH_2_ and CB18crown6/SBA-15 did not change much (Figure 4b,c). In addition, a well-ordered parallel mesochannel could also be identified, even when the APTES and CB18crown6 were attached to the SBA-15, as shown in Figure 5e,f.

Figure 5a,b show TEM images of the SBA-15-Ex and the CB18crown6/SBA-15, respectively. The TEM image revealing the well-ordered mesoporous channels of the SBA-15 was well maintained even after modification with CB18crown6. In addition, combined with the BET data that will be discussed later, the TEM images may also indicate that the diameter of the channels of CB18crown6/SBA-15 was slightly lower than that of SBA-15-Ex. This result confirms that the CB18crown6/SBA-15 adsorbent with uniform mesopores was successfully prepared, which will be beneficial to the performance of the adsorbent.

#### 3.1.5. Nitrogen Adsorption–Desorption Analysis

**Figure 6:**
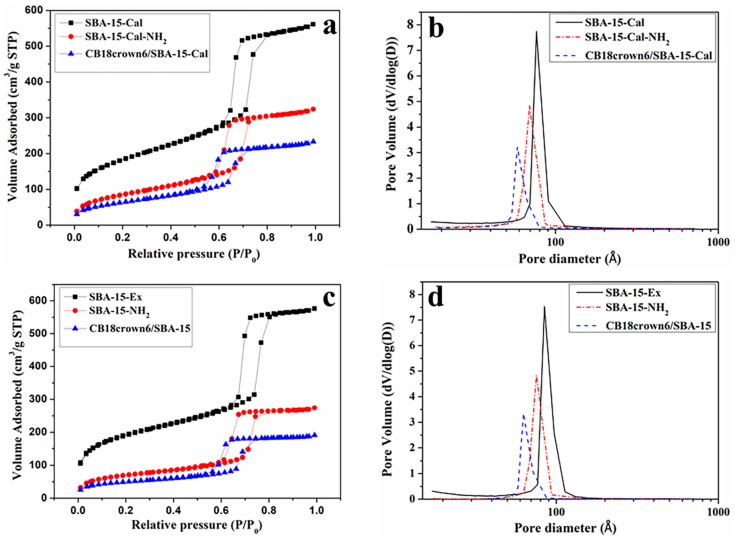
N_2_ adsorption–desorption isotherms and pore size distributions of (**a**,**b**) SBA-15-Cal, SBA-15-Cal-NH_2_ and CB18crown6/SBA-15-Cal; (**c**,**d**) SBA-15-Ex, SBA-15-NH_2_, and CB18crown6/SBA-15. (STP: Standard Temperature and Pressure).

The N_2_ adsorption–desorption isotherms of the SBA-15-Cal, SBA 15-Cal-NH_2_, CB18crown6/SBA-15-Cal, SBA-15-Ex, SBA 15-NH_2_, and CB18crown6/SBA-15 were measured, and the results are shown in Figure 6 and summarized in Table 1. All of the materials exhibited type-IV isotherms with H1-type hysteresis loops, indicating the presence of a mesoporous structure. Figure 6a shows the N_2_ adsorption–desorption isotherms of SBA-15-Cal, SBA 15-Cal-NH_2_, and CB18crown6/SBA-15-Cal (SBA-15 obtained via calcination). In the N_2_ adsorption–desorption isotherms, the isotherm of SBA-15-Cal was shifted to the left after modification with CB18crown6. This means that the CB18crown6/SBA-15-Cal has a relatively small volume and small pore size compared to the SBA-15-Cal-NH_2_ and the SBA-15-Cal [23,30]. Figure 6c shows the N_2_ adsorption–desorption isotherms of SBA-15-Ex, SBA 15-NH_2_, and CB18crown6/SBA-15. Similar adsorption behavior was observed; however, the surface area and pore size of the SBA-15-Ex are larger than those of the SBA-15-Cal (Table 1). This may be due to the fact that when P123 is removed at high temperatures by calcination, the silanol groups on the surface may be combined with another silanol group by heat, leading to decreased pore size. Furthermore, it was found that the volume reduction of SBA-15-Ex is greater than that of SBA-15-Cal after modification with APTES and CB18crown6 under the same conditions. These phenomena not only confirm the modification of CB18crown6/SBA-15 via covalent grafting, but also indicate that the SBA-15-Ex could introduce more APTES and CB18crown6, depending on the presence of more silanol groups on the SBA-15 [31]. This is consistent with the reasons we mentioned earlier for choosing SBA-15-Ex for further modification. In addition, even after surface modification, the specific surface area of CB18crown6/SBA-15 can still reach 172 m^2^/g.

### 3.2. Adsorption Performance and Selective Adsorption

**Figure 7:**
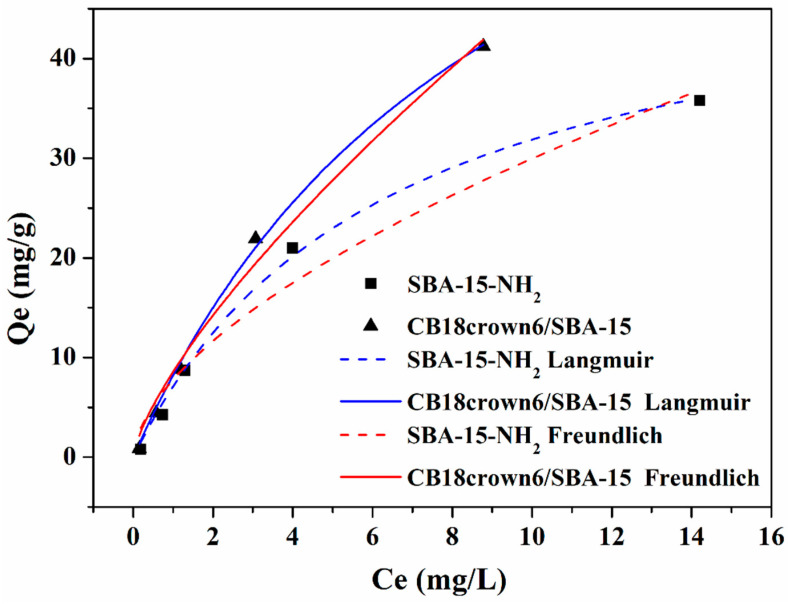
Adsorption isotherms of the Langmuir and Freundlich models for Cr(VI) adsorption by SBA-15-NH_2_ and CB18crown6/SBA-15.

Before the selective adsorption experiment, we checked first whether the CB18crown6 modification of SBA-15 could effectively improve the adsorption performance. The adsorption isotherm was studied for SBA-15-NH_2_ and CB18crown6/SBA-15. Figure 7 displays the adsorption isotherms of Cr(VI) adsorbed onto CB18crown6/SBA-15 at pH 2 for 24 h. Figure 7 demonstrates that the Cr(VI) adsorption capacity of CB18crown6/SBA-15 was enhanced after surface functionalization of SBA-15-NH_2_ with CB18crown6, though the data may not be representative of the maximum experimental adsorption capacity. This may be attributable to the synergy of the –NH– and crown ether groups. In order to obtain more precise information on the adsorption capacity, further works should be conducted on the kinetics of metal ions’ adsorption in the present multicomponent solution systems, as well as true maximum adsorption capacity at longer times and different temperatures. Although the adsorption capacity of CB18crown6/SBA-15 and SBA-15-NH_2_ was similar at low concentrations of Cr(VI) (Ce = 1 mg/L or less), CB18crown6/SBA-15 showed higher adsorption capacity of Cr(VI) than SBA-15-NH_2_ at higher concentrations (Ce = 2 mg/L or more). This result may be attributable to the different affinity of CB18crown6 and the amine (–NH_2_) group for Cr(VI). Based on these results, therefore, CB18crown6/SBA-15 would be a more useful adsorbent than SBA-15-NH_2_ at higher Cr(VI) concentrations. The Langmuir and Freundlich isotherms were used to evaluate the processes of Cr(VI) adsorption onto SBA-15-NH_2_ and CB18crown6/SBA-15. The Langmuir isotherm of the homogeneous system illustrates the single-layer adsorption behavior. The Freundlich isotherm explains the multilayer adsorption and heterogeneous adsorbent systems. The nonlinear Langmuir model Equations (2) and (3), and the Freundlich model Equation (4), are expressed as follows [26,32]:

(2) $$q_{e}=\frac{q_{m}{\mathrm{KC}}_{e}}{1+{\mathrm{KC}}_{e}}$$

(3) $$R_{L}=\frac{1}{1+{\mathrm{KC}}_{0}}$$

(4) $$q_{e}=K_{f}C_{e}{}^{1/n}$$

where q_e_ (mg/g) and q_m_ (mg/g) are the amount of Cr(VI) adsorbed on SBA-15-NH_2_ and CB18crown6/SBA-15 at the equilibrium and the maximum adsorption capacity, respectively, Ce (mg/L) is the equilibrium concentration of Cr(VI), K (L/mg) is the Langmuir constant, K_f_ represents the Freundlich constant—which is an indicator of adsorption capacity—and 1/n is the adsorption strength of the system.

The fitting results are shown in Figure 7 and Table 2, which clearly illustrate that the fitting results from the Langmuir isothermal adsorption model (R^2^ = 0.996 and 0.998) were more suitable than those from the Freundlich model (R^2^ = 0.963 and 0.987). This result suggests the homogeneous adsorption of Cr(VI) ions by the SBA-15-NH_2_ and CB18crown6/SBA-15. The maximum adsorption capacity obtained from the Langmuir isotherm for the adsorption of Cr(VI) onto SBA-15-NH_2_ was 52.1 mg/g, while the maximum adsorption capacity of CB18crown6/SBA-15 was 86.0 mg/g. The adsorption performance was compared with related adsorbents in previous works (Table 3) [33,34,35,36,37,38,39,40,41], showing that the adsorption performance of CB18crown6/SBA-15 is generally comparable to other adsorbents.

**Figure 8:**
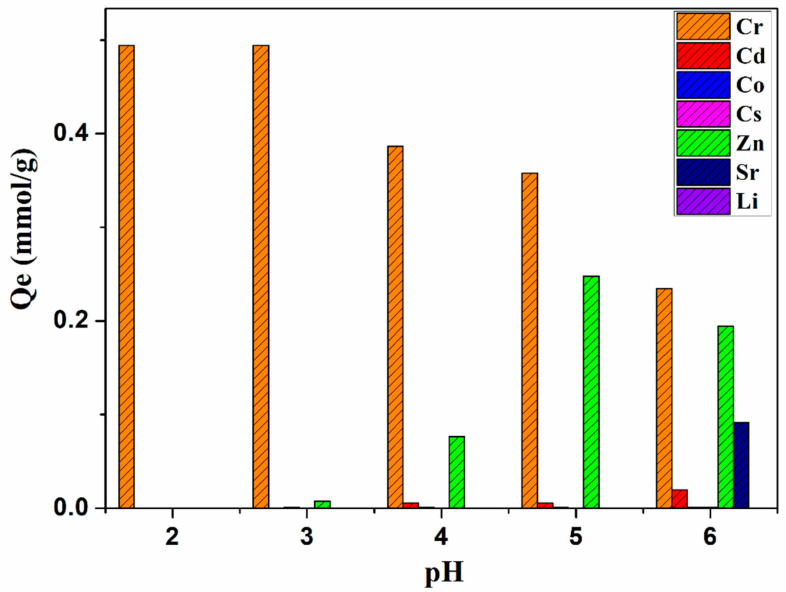
Adsorption of various metal ions from the 0.5 mmol/L mixed solutions of Cr, Cd, Co, Cs, Zn, Sr, and Li onto CB18crown6/SBA-15.

**Figure 9:**
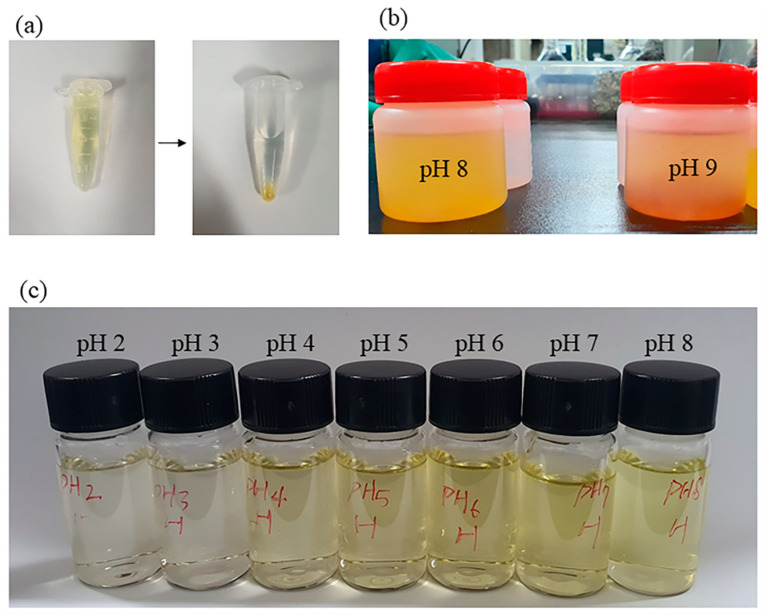
The optical images of the solution after selective adsorption at different pH levels (**a**): Before and after adsorption (Separation of adsorbent and metal ions solution by centrifugation) at pH 2; (**b**): Metal ion solution was precipitated under alkaline conditions; (**c**): Multi-metal-ion mixture after testing for the selective adsorption of metal ions under different pH conditions).

To further study the practical applicability of CB18crown6/SBA-15, the adsorption efficiency of the adsorbent for metal ions in the aqueous solutions containing seven metal ions—chromium, zinc, lithium, cadmium, cobalt, strontium, and cesium ions—was measured. Competitive metal ion adsorption was performed in the pH range 2–6 in a batch experiment with a mixture of all seven metal ions at concentrations of 0.5 mmol/L of each metal ion at 25 °C. The removal efficiency of each metal ion from the mixed solution is shown in Figure 8. Cr(VI) showed high adsorption at pH 2 and 3, while at pH 5–6, Zn(II) adsorption was also detected. However, other metal ions such as Co, Cs, Cs, Sr, and Li ions were not adsorbed or marginally adsorbed. In addition, above pH 7–8, it was difficult to measure adsorption accurately, because the metal ions were precipitated (Figure 9b); for example, Zn^2+^, Cd^2+^, and Co^2+^ may form metal hydroxides or salts under alkaline conditions.

This experimental result indicates that CB18crown6/SBA-15 exhibits effectively selective adsorption of Cr(VI) at pH 2. This may be attributable to the form of Cr(VI) at this pH level. HCrO_4_^−^ and Cr_2_O_7_^2−^ are usually found at pH 2–6 [42]. According to previous reports, when other crown ethers are used to adsorb Cr(VI), the adsorption of Cr(VI) can be effectively promoted under acidic conditions; the possible mechanism can be explained by Equation (5) [43]. In addition, since the –NH– group still exists after the CB18crown6 is attached, the –NH_2_^+^– protonated from –NH– under acidic conditions may also promote the adsorption of Cr(VI); the possible mechanism can be explained by Equation (6) [44,45].

H^+^ + Crown ether + HCrO_4_^−^ → (H crown ether^+^)(HCrO_4_^−^) (5)

–NH_2_^+^– + HCrO_4_^−^ → –NH_2_^+^–…HCrO_4_^−^ (6)

As the pH increases, the H^+^ content in the solution decreases, and the surface charge and properties of the adsorbent change, so the corresponding Cr(VI) adsorption capacity decreases. The adsorption of metal ions was also checked using optical images (Figure 9a,c). The image (Figure 9c) shows the multi-metal-ion mixture after testing for the selective adsorption of metal ions, which displays a different color due to the difference in chromium adsorption performance under different pH conditions.

**Scheme 2:**
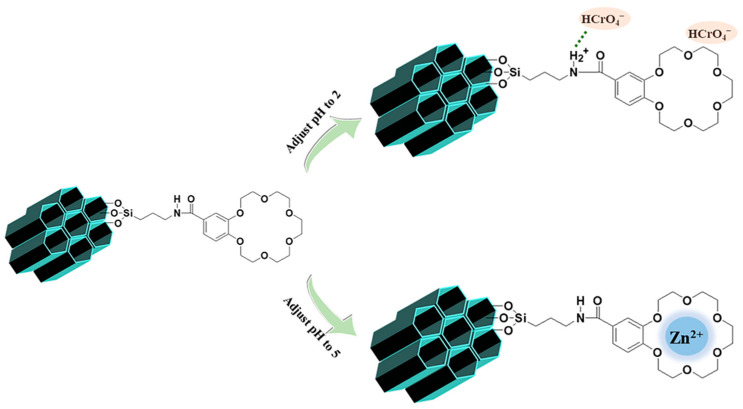
Possible mechanism of adsorption of Cr(VI) and Zn(II) by CB18crown6/SBA-15.

In addition, when the pH increased, selective adsorption of zinc ions was also observed. Deb et al. [46] reported that the poly(methyl acrylate) (PMA) resin functionalized with dibenzo-18-crown-6 (DB18C6) showed the binding of zinc ions to the oxygen ring of the crown ether. Thus, the CB18crown6/SBA-15 was able to adsorb zinc ions with the same number of oxygen rings in the crown ether. The diameter of the CB18crown6 in this work was ~2.6–3.2 Å [47]. The diameter of zinc ions is 1.48 Å, which is relatively smaller than that of CB18crown6, and seems not to match with the latter. However, according to previous reports, although zinc ions are too small to fit into a single cavity, there is a tendency for Zn(II) ions to form a “sandwich’’-type complex with adjacent crown units of CB18crown6/SBA-15 [48]. After grafting CB18crown6 onto the surface of SBA-15-NH_2_, the mesoporous surface contains a large number of crown ether groups—especially adjacent crown ether groups—so that Zn(II) ions are complexed by CB18crown6 in a sandwich-type complex and form the crown ether–metal ion complex with a 2:1 ratio, leading to selective adsorption of zinc ions. The adsorption capacities of other metal ions were very small or did not show adsorption performance, indicating that their diameter does not matching the cavities of CB18crown6 (for example: the sizes of these ions are Cd^2+^ ~1.94 Å, Li^+^ ~1.2 Å, Cs^+^ ~3.8 Å, and Sr^2+^ ~2.36 Å) [49]. Despite the diameter of Co^2+^ (1.44 Å) being similar to that of Zn^2+^ ions, according to the previous research carried out in our laboratory, Zn^2+^ is believed to diffuse in silicon mainly via an interstitial–substitutional mechanism, where this interstitial–substitutional-diffusing Zn^2+^ regularly “kicks out” a Si atom and assumes a substitutional position in the course of the diffusion process. Therefore, it exhibits strong selective adsorption of Zn^2+^, even in multicomponent metal solutions containing Ni^2+^, Li^+^, Co^2+^, Cd^2+^, and Zn^2+^ [50]. With regard to the adsorption selectivity of CB18crown6/SBA-15 for Zn(II) metal ions, in addition to the extent to which the ion size matches that of the CB18crown6 cavities, the binding energy and Gibbs free energy difference of the hydration process for these ions also play a major role in the selectivity, and this merits further investigation. As Deb et al. [46] reported, the substantial negative values of binding energy as well as Gibbs free energy indicate that the crown-ether-appended polymeric resin exhibits excellent adsorption performance for Zn(II) ions [51]. In addition, it may also be slightly negatively charged under roughly neutral pH conditions, which is favorable for the removal of positively charged Zn(II), while under lower pH conditions, H^+^ may compete with Zn(II); thus, as the pH decreases to 2, its adsorption performance weakens. The possible mechanism of adsorption of Cr(VI) and Zn(II) by CB18crown6/SBA-15 is illustrated in Scheme 2. CB18crown6/SBA-15 mainly shows the removal of Cr(VI) and Zn(II) at different pH levels. At pH 6, CB18crown6/SBA-15 shows a small amount of adsorption of Sr^2+^. The adsorption of Sr^2+^ onto CB18crown6/SBA-15 is also possible through complexation of the oxygen atom inside CB18crown6/SBA-15 with Sr^2+^. As discussed above, the diameter of Sr^2+^ is 2.36 Å—larger than that of Cd^2+^, Co^2+^, and Li^+^—while the diameter of Cs^+^ is larger than that of CB18crown6. Therefore, Sr^2+^ is relatively easy to complex with CB18crown6 compared to the ions mentioned above, leading to a display of marginal adsorption performance.

In this work, we focused on the adsorption behavior of metal ions—including Cr(VI) ions—in a multicomponent solution system. Though we used a multicomponent system containing seven coexisting metal ions (cations: Zn^2+^, Cd^2+^, Co^2+^, Cs^+^, Sr^2+^, and Li^+^; and anions: Cr^6+^), it would be necessary for future works to compare their adsorption with other anions—such as Cl^−^, NO_3_^−^, SO_4_^2−^, and PO_4_^3−^, etc.—or even to compare the cations with other common ones present in water, such as Na^+^, K^+^, and Ca^2+^, etc., in order to better understand the adsorption behavior of Cr(VI).

### 3.3. Reusability

**Figure 10:**
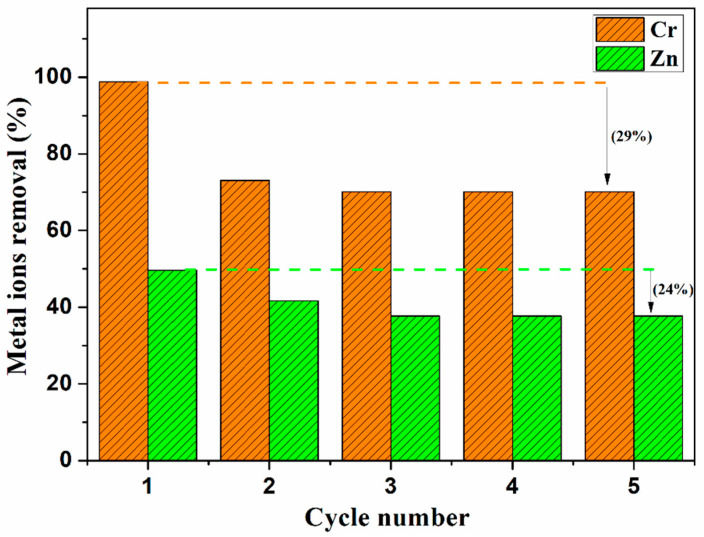
Reusability of CB18crown6/SBA-15 for adsorption of Cr(VI) and Zn(II).

The recyclability of the adsorbent is very important for the its practical application. To test its recyclability, CB18crown6/SBA-15 was evaluated through Cr(VI) and Zn(II) adsorption/desorption cycles. CB18crown6/SBA-15 was immersed in a 10 mL aqueous solution of a single metal ion (0.5 mmol/L). After each adsorption step, the CB18crown6/SBA-15 sample was regenerated by stirring Cr(VI)-loaded CB18crown6/SBA-15 in 0.1 mol/L NaOH for 1 h and Zn(II)-loaded CB18crown6/SBA-15 in 0.1 mol/L HCl for 1 h. After centrifugation and washing with deionized water, the samples were vacuum-dried at 70 °C for 12 h. Regenerated CB18crown6/SBA-15 was then used for the next metal ion adsorption cycle. The adsorption and regeneration cycles were repeated five times. Figure 10 shows the results of recycling test, clearly showing that the adsorbent can still reach a recovery efficiency of 71 and 76% for Cr(VI) and Zn(II), respectively, after being recycled five times. The decrease in adsorption efficiency can be ascribed to the inescapable mass loss during the adsorption–desorption processes and the partial, non-complete regeneration of the adsorbent [52]. This result indicates that CB18crown6/SBA-15 is promising for the practical removal of Cr(VI) and Zn(II).

## 4. Conclusions

In this study, crown-ether-modified SBA-15 (CB18crown6/SBA-15) was facilely prepared by introducing 4′-carboxybenzo-18-crown-6 (CB18crown6) onto a mesoporous silica support (SBA-15) through covalent attachment. Physicochemical, surface, and morphological characterizations revealed the successful preparation of the adsorbent. Batch adsorption experiments showed that the optimal conditions for Cr(VI)-selective adsorption were observed at pH 2, while the optimal conditions for Zn(II)-selective adsorption were observed at pH 5, from the mixed aqueous solutions of chromium, zinc, lithium, cadmium, cobalt, strontium, and cesium ions using CB18crown6/SBA-15. The process of adsorption of Cr(VI) by CB18crown6/SBA-15 was best explained by the Langmuir adsorption isotherm. In addition, the recycling tests showed that the CB18crown6/SBA-15 was able to achieve 71% reuse efficiency of Cr(VI) and 76% reuse efficiency of Zn(II), even after five recycles, confirming that CB18crown6/SBA-15 can be used as a highly stable and recyclable adsorbent. Therefore, it can be summarized that the development of novel hybrid mesoporous-silica-based adsorbent materials such as CB18crown6/SBA-15, with improved adsorption efficiency, is highly desirable.

## Figures and Tables

**Table 1:** Textural properties of SBA-15-Cal, SBA-15-Cal-NH_2_, CB18crown6/SBA-15-Cal, SBA-15-Ex, SBA-15-NH_2_, and CB18crown6/SBA-15.

Samples	Surface Area (m^2^/g)	Pore Volume (cm^3^/g)	Pore Size (nm)
SBA-15-Cal	640	0.9	7.7
SBA-15-Cal-NH_2_	311	0.5	6.9
CB18crown6/SBA-15-Cal	232	0.4	5.9
SBA-15-Ex	652	0.9	8.5
SBA-15-NH_2_	246	0.4	7.6
CB18crown6/SBA-15	172	0.3	6.3

**Table 2:** Parameters associated with the Langmuir and Freundlich isotherms for the adsorption of Cr(VI) onto SBA-15-NH_2_ and CB18crown6/SBA-15.

Adsorbent	Langmuir Isotherm			Freundlich Isotherm		
	q_m_ (mg/g)	K (L/mg)	R^2^	K_f_ (mg/g (L/mg)^1/n^)	n	R^2^
SBA-15-NH_2_ CB18crown6/SBA-15	52.1	0.1575	0.996	7.76	1.7039	0.963
	86.0	0.1057	0.998	8.58	1.3699	0.987

**Table 3:** Comparative studies of the Cr(VI) adsorption capacity of CB18crown6/SBA-15 with other related sorbents.

Adsorbents *	Adsorbates	Adsorption Capacity (mg/g)	References
BC ^1^	Cr(VI)	21.3	[33]
4-VP/HEMA-grafted PET ^2^	Cr(VI)	81.0	[34]
PyR resin ^3^	Cr(VI)	94.3	[35]
SGE-10/12-en ^4^	Cr(VI)	109.7	[36]
AFC-coated silica gel ^5^	Cr(VI)	65.0	[37]
SBA/SA ^6^	Cr(VI)	66.7	[38]
SBA-TMPED ^7^	Cr(VI)	55.0	[39]
PPy/SBA-15 ^8^	Cr(VI)	194.2	[40]
PD/MCM-41 ^9^	Cr(VI)	70.9	[41]
CB18crown6/SBA-15	Cr(VI)	86.0	This work

* (Note: a brief explanation of the above samples is summarized as follows). ^1^ BC: wheat-residue-derived black carbon; ^2^ 4-VP/HEMA-grafted PET: 4-vinly pyridine (4-VP) and 2-hydroxyethylmethacrylate (HEMA) monomer graft onto poly(ethylene terephthalate) (PET) fibers; ^3^ PyR resin: 4-vinylpyridine:divinylbenzene copolymer of gel structure with 2-chloroacetamide; ^4^ SGE-10/12-en: functionalization of poly(glycidyl methacrylate-co-ethylene glycol dimethacrylate) with ethylene diamine; ^5^ AFC-coated silica gel: aniline-formaldehyde-condensate-coated silica gel; ^6^ SBA/SA: SBA-15 mesoporous materials modified with N-propylsalicylaldimine groups; ^7^ SBA-TMPED: SBA-15 functionalized with N-[3-(trimethoxysilyl)propyl]-ethylenediamine (TMPED); ^8^ PPy/SBA-15: monodisperse polypyrrole/SBA-15; ^9^ PD/MCM-41: MCM-41 silica coated on the processed diatomite.

## Data Availability

The data presented in this study are available on request from corresponding author, upon reasonable request.

## References

[1] Fu F.L., Wang Q. (2011). Removal of heavy metal ions from wastewaters: A review. J. Environ. Manag..

[2] Agarwal M., Singh K. (2017). Heavy metal removal from wastewater using various adsorbents: A review. J. Water Reuse Desalination.

[3] Zhang M.Y., Song L.H., Jiang H.F., Li S., Shao Y.F., Yang J.Q., Li J.F. (2017). Biomass based hydrogel as an adsorbent for the fast removal of heavy metal ions from aqueous solutions. J. Mater. Chem. A.

[4] Barnabas M.J., Parambadath S., Mathew A., Park S.S., Vinu A., Ha C.S. (2016). Highly efficient and selective adsorption of In3þ on pristine Zn/Al layered double hydroxide (Zn/Al-LDH) from aqueous solutions. J. Solid State Chem..

[5] Xie Y.Q., Lin J., Liang J., Li M.H., Fu Y.W., Wang H.T., Tu S., Li J. (2019). Hypercrosslinked mesoporous poly(ionic liquid)s with high density of ion pairs: Efficient adsorbents for Cr(VI) removal via ion-exchange. Chem. Eng. J..

[6] Njoya O., Zhao S.X., Qu Y.F., Shen J.M., Wang B.Y., Shi H., Chen Z.L. (2020). Performance and potential mechanism of Cr(VI) reduction and subsequent Cr(III) precipitation using sodium borohydride driven by oxalate. J. Environ. Manag..

[7] Lu W.L., Duan C., Zhang Y.L., Gao K., Dai L., Shen M.X., Wang W.L., Wang J., Ni Y.H. (2021). Cellulose-based electrospun nanofiber membrane with core-sheath structure and robust photocatalytic activity for simultaneous and efficient oil emulsions separation, dye degradation and Cr(VI) reduction. Carbohydr. Polym..

[8] Aguado J., Arsuaga J.M., Arencibia A., Lindo M., Gascón V. (2009). Aqueous heavy metals removal by adsorption on amine-functionalized mesoporous silica. J. Hazard. Mater..

[9] Alothman Z.A., Apblett A.W. (2010). Metal ion adsorption using polyamine-functionalized mesoporous materials prepared from bromopropyl-functionalized mesoporous silica. J. Hazard. Mater..

[10] Elwakeel K.Z., El-Sayed G.O., Darweesh R.S. (2013). Fast and selective removal of silver(I) from aqueous media by modified chitosan resins. Int. J. Miner. Process..

[11] Li G.L., Zhao Z.S., Liu J.Y., Jiang G.B. (2011). Effective heavy metal removal from aqueous systems by thiol functionalized magnetic mesoporous silica. J. Hazard. Mater..

[12] Verma M.L., Rani V. (2021). Biosensors for toxic metals, polychlorinated biphenyls, biological oxygen demand, endocrine disruptors, hormones, dioxin, phenolic and organophosphorus compounds: A review. Environ. Chem. Lett..

[13] Narayan R., Nayak U.Y., Raichur A.M., Garg S. (2018). Mesoporous Silica Nanoparticles: A Comprehensive Review on Synthesis and Recent Advances. Pharmaceutics.

[14] Suib S.L. (2017). A Review of Recent Developments of Mesoporous Materials. Chem. Rec..

[15] Mureseanu M., Reiss A., Stefanescu I., David E., Parvulescu V., Renard G., Hulea V. (2008). Modified SBA-15 mesoporous silica for heavy metal ions remediation. Chemosphere.

[16] Shahbazi A., Younesi H., Badiei A. (2011). Functionalized SBA-15 mesoporous silica by melamine-based dendrimer amines for adsorptive characteristics of Pb(II), Cu(II) and Cd(II) heavy metal ions in batch and fixed bed column. Chem. Eng. J..

[17] Duman O., Ayranci E. (2010). Attachment of benzo-crown ethers onto activated carbon cloth to enhance the removal of chromium, cobalt and nickel ions from aqueous solutions by adsorption. J. Hazard. Mater..

[18] Awual M.R. (2016). Ring size dependent crown ether based mesoporous adsorbent for high cesium adsorption from wastewater. Chem. Eng. J..

[19] Hong M.Z., Wang X., You W.J., Zhuang Z.Y., Yu Y. (2017). Adsorbents based on crown ether functionalized composite mesoporous silica for selective extraction of trace silver. Chem. Eng. J..

[20] Luo H., Dai S., Bonnesen P.V., Haverlock T.J., Moyer B.A., Buchanan III A.C. (2006). A Striking Effect of Ionic-Liquid Anions in the Extraction of Sr^2+^ and Cs^+^ by Dicyclohexano-18-Crown-6. Solvent Extr. Ion Exch..

[21] Mohamed M.G., Kuo S.W. (2020). Crown Ether-Functionalized Polybenzoxazine for Metal Ion Adsorption. Macromolecules.

[22] Wang W.H., Gong C., Wang W.C., Kong F.T., Kim H.C., Fullerton-Shirey S.K., Seabaugh A., Cho K. (2017). Energetics of metal ion adsorption on and diffusion through crown ethers: First principles study on two-dimensional electrolyte. Solid State Ion.

[23] Parambadath S., Mathew A., Barnabas M.J., Kim S.Y., Ha C.S. (2016). Concentration-dependant selective removal of Cr(III), Pb(II) and Zn(II) from aqueous mixtures using 5-methyl-2-thiophenecarboxaldehyde Schiff base-immobilised SBA-15. J. Sol-Gel Sci. Technol..

[24] Bérube F., Kaliaguine S. (2008). Calcination and thermal degradation mechanisms of triblock copolymer template in SBA-15 materials. Microporous Mesoporous Mater..

[25] De Avila S.G., Silva L.C.C., Matos J.R. (2016). Optimisation of SBA-15 properties using Soxhlet solvent extraction for template removal. Microporous Mesoporous Mater..

[26] Yan Y.Z., Nagappan S., Yoo J.M., Nechikkattu R., Park S.S., Ha C.S. (2021). Polyethyleneimine-grafted polysilsesquioxane hollow spheres for the highly efficient removal of anionic dyes and selective adsorption of Cr(VI). J. Environ. Chem. Eng..

[27] Huang J., Ye M., Qu Y.Q., Chu L.F., Chen R., He Q.Z., Xu D.F. (2012). Pb (II) removal from aqueous media by EDTA-modified mesoporous silica SBA-15. J. Colloid Interface Sci..

[28] Wu H.M., Xiao Y., Guo Y., Miao S.J., Chen Q.Q., Chen Z. (2020). Functionalization of SBA-15 mesoporous materials with 2-acetylthiophene for adsorption of Cr(III) ions. Microporous Mesoporous Mater..

[29] Dana E., Sayari A. (2012). Adsorption of heavy metals on amine-functionalized SBA-15 prepared by co-condensation: Applications to real water samples. Desalination.

[30] Yang J.P., Shen D.K., Wei Y., Li W., Zhang F., Kong B., Zhang S.H., Teng W., Fan J.W., Zhang W.X. (2015). Monodisperse core-shell structured magnetic mesoporous aluminosilicate nanospheres with large dendritic mesochannels. Nano Res..

[31] Albayati T.M., Salih I.K., Alazzawi H.F. (2019). Synthesis and characterization of a modified surface of SBA-15 mesoporous silica for a chloramphenicol drug delivery system. Heliyon.

[32] Ge F., Li M.M., Ye H., Zhao B.X. (2012). Effective removal of heavy metal ions Cd^2+^, Zn^2+^, Pb^2+^, Cu^2+^ from aqueous solution by polymer-modified magnetic nanoparticles. J. Hazard. Mater..

[33] Wang X.S., Chen L.F., Li F.Y., Chen K.L., Wan W.Y., Tang Y.J. (2010). Removal of Cr (VI) with wheat-residue derived black carbon: Reaction mechanism and adsorption performance. J. Hazard. Mater..

[34] Yigitoglu M., Arslan M. (2009). Selective removal of Cr(VI) ions from aqueous solutions including Cr(VI), Cu(II) and Cd(II) ions by 4-vinly pyridine/2-hydroxyethylmethacrylate monomer mixture grafted poly(ethylene terephthalate) fiber. J. Hazard. Mater..

[35] Neagu V. (2009). Removal of Cr(VI) onto functionalized pyridine copolymer with amide groups. J. Hazard. Mater..

[36] Nastasović A., Sandić Z., Suručić L., Maksin D., Jakovljević D., Onjia A.J. (2009). Kinetics of hexavalent chromium sorption on amino-functionalized macroporous glycidyl methacrylate copolymer. J. Hazard. Mater..

[37] Kumar P.A., Ray M., Chakraborty S. (2007). Hexavalent chromium removal from wastewater using aniline formaldehyde condensate coated silica gel. J. Hazard. Mater..

[38] Dindar M.H., Yaftian M.R., Rostamnia S. (2015). Potential of functionalized SBA-15 mesoporous materials for decontamination of water solutions from Cr(VI), As(V) and Hg(II) ions. J. Environ. Chem. Eng..

[39] Dobrzynska J. (2021). Amine- and thiol-functionalized SBA-15: Potential materials for As(V), Cr(VI) and Se(VI) removal from water. Comparative study. J. Water Process. Eng..

[40] Wang L., Gao P., Liu M.X., Huang Z.Q., Lan S.X., Li Y., Rao W.H., Liu Y.L., Du R., Yu C.B. (2021). The fabrication of monodisperse polypyrrole/SBA-15 composite for the selective removal of Cr(VI) from aqueous solutions. New J. Chem..

[41] Selim A.Q., Mohamed E.A., Mobarak M., Zayed A.M., Seliem M.K., Komarneni S. (2018). Cr(VI) uptake by a composite of processed diatomite with MCM-41: Isotherm, kinetic and thermodynamic studies. Microporous Mesoporous Mater..

[42] Saha B., Orvig C. (2010). Biosorbents for hexavalent chromium elimination from industrial and municipal effluents. Coord. Chem. Rev..

[43] Parinejad M., Yaftian M.R. (2007). A study on the removal of chromium (VI) oxanions from acid solutions by using oxonium ion-crown ether complexes as mobile carrier agents. Iran. J. Chem. Chem. Eng..

[44] Qiu B., Guo J., Zhang X., Sun D.Z., Gu H.B., Wang Q., Wang H.W., Wang X.F., Zhang X., Weeks B.L. (2014). Polyethylenimine Facilitated Ethyl Cellulose for Hexavalent Chromium Removal with a Wide pH Range. ACS Appl. Mater. Interfaces.

[45] Bao S.Y., Yang W.W., Wang Y.J., Yu Y.S., Sun Y.Y., Li K.F. (2020). PEI grafted amino-functionalized graphene oxide nanosheets for ultrafastand high selectivity removal of Cr(VI) from aqueous solutions by adsorption combined with reduction: Behaviors and mechanisms. Chem. Eng. J..

[46] Deb A.K.S., Sahu P., Boda A., Ali S.M., Shenoy K.T., Upadhyay D. (2020). DFT and MD simulation supplemented experiments for isotopic fractionation of zinc compounds using a macrocyclic crown ether appended polymeric resin. Phys. Chem. Chem. Phys..

[47] Shi L., Xu A., Cheng Y.H. (2019). Ether-Group-Mediated Aqueous Proton Selective Transfer across Graphene-Embedded 18-Crown-6 Ether Pores. J. Phys. Chem. C.

[48] Gao B.J., Wang S.W., Zhang Z.G. (2010). Study on complexation adsorption behavior of dibenzo-18-crown-6 immobilized on CPVA microspheres for metal ions. J. Incl. Phenom. Macrocycl. Chem..

[49] Shannon R.D. (1976). Revised effective ionic radii and systematic studies of interatomic distances in halides and chalcogenides. Acta Crystallogr. A.

[50] Mathew A., Parambadath S., Kim S.Y., Ha H.M., Ha C.S. (2016). Diffusion mediated selective adsorption of Zn^2+^ from artificial seawater by MCM-41. Microporous Mesoporous Mater..

[51] Mututuvari T.M., Tran C.D. (2014). Synergistic adsorption of heavy metal ions and organic pollutants by supramolecular polysaccharide composite materials from cellulose, chitosan and crown ether. J. Hazard. Mater..

[52] Yan Y.Z., An Q.D., Xiao Z.Y., Zhai S.R., Zhai B., Shi Z. (2017). Interior multi-cavity/surface engineering of alginate hydrogels with polyethylenimine for highly efficient chromium removal in batch and continuous aqueous systems. J. Mater. Chem. A.

